# Social Media Accounts of Food and Beverage Brands Have Disproportionately More Black and Hispanic Followers than White Followers

**DOI:** 10.1089/heq.2020.0068

**Published:** 2021-06-15

**Authors:** Pasquale E. Rummo, Josh H. Arshonsky, Andrea L. Sharkey, Omni L. Cassidy, Marie A. Bragg

**Affiliations:** ^1^ Department of Population Health, New York University School of Medicine, New York, New York, USA.; ^2^ NYU School of Global Public Health, New York, New York, USA.

**Keywords:** racially targeted marketing, social media, sugar-sweetened beverages, fast food, policy

## Abstract

**Introduction:** On television, food companies promote their least nutritious products to Black and Hispanic youth more than White youth, but little is known about the extent to which Black and Hispanic adolescents may disproportionately engage with unhealthy food and beverage brands on social media relative to White adolescents.

**Methods:** In 2019, we purchased and analyzed demographic data of social media users who followed 27 of the most marketed food/beverage brands on Instagram and Twitter. We used one-sample *t*-tests to compare percentages of Black, Hispanic, and White followers of the selected brands' accounts versus all social media accounts, and independent samples *t*-tests to compare followers of sugary versus low-calorie drink brands. We also used linear regression to examine associations between racially targeted marketing practices and the percentages of Black, Hispanic, and White followers on social media.

**Results:** On Instagram, the percentage of Black followers of the selected brands (12.7%) was higher than the percentage of Black followers of any account (7.8%) (*p*<0.001). On Twitter, findings were similar for Hispanic users but opposite for White users. A higher racially targeted ratio was positively associated with the percentage of Black followers, and negatively associated with the percentage of White followers. Sugary drink brands had more Hispanic followers than low-calorie drink brands (*p*<0.001).

**Conclusions:** Unhealthy food/beverage brands that target Black adolescents have a disproportionately higher percentage of Black followers on social media relative to White followers. These findings support the 2019 proposal to restrict racially targeted advertising through the Children's Online Privacy and Protection Act.

## Introduction

The National Academy of Medicine has identified food advertisements (“ads”) as a major driver of poor diet and childhood obesity.^1–6^ In the United States, Black and Hispanic youth are disproportionately targeted with food and beverage ads that promote unhealthy foods and beverages.^7^ In 2017, food companies spent nearly $13 billion on food ads across all platforms (e.g., television [TV] and social media), including $787 million on Hispanic-targeted TV ads and $333 million on Black-targeted TV ads.^8^ Between 2013 and 2017, spending on Black-targeted TV ads increased by 50%.^8^

These racially targeted marketing practices may reflect companies' recognition of the cultural and financial power of communities of color, but targeted marketing can be problematic for public health when companies promote products that contribute to disparities in diet-related health outcomes.^9^ Food companies have identified Black youth as a valuable market segment because of their high media use, spending patterns, and role as cultural trendsetters.^9^ However few studies have examined food advertising exposure among Black and Hispanic communities, and most of those studies have focused on TV or outdoor ads.^9–15^

Although TV advertising remains the most popular promotional channel, social media is growing rapidly.^12^ In 2018, almost all adolescents reported that they own a smartphone, and 90% of adolescents use social media.^11^ The most frequently used sites among adolescents are YouTube (85%), SnapChat (69%), Instagram (72%), Facebook (51%), and Twitter (32%), and most adolescents report using multiple platforms.^16^ Companies now dedicate substantial portions of their marketing expenditures to social media.^17^ There was ∼570% increase in social media account creation by food and beverage companies between 2007 and 2016.^18^ These online platforms provide companies with the opportunity to more precisely track and interact with potential consumers through previously unavailable methods, such as geo-location targeting and creating free accounts that users can “follow” (e.g., @Sprite).^19,20^

Following a food and beverage brand account allows users to engage with brands by “liking,” commenting, or sharing the company's posts with others in their social network. This level of engagement is unique to social media marketing and increases companies' access to consumers, particularly adolescents.^21,22^ Although empirical data are limited, market research suggests that learning about new products is the most common reason consumers engage with brands on social media.^23^

Business marketing literature indicates that brand engagement affects purchase intent and actual purchases.^24–26^ In one study commissioned by Twitter, positive Tweets that increased likelihood of “sharing” were nine times more effective in increasing video game sales compared with traditional advertising. Another study examining social media metrics and music album sales found that following music artists on social media was positively associated with increases in album sales.^27^ There are no published studies examining how engaging with food and beverage brands on social media influences purchase intent or actual purchases among Black or Hispanic youth. Exposure to targeted ads on social media, however, is likely designed to increase sales of advertised unhealthy products. Sales, and ultimately consumption, of these products may contribute to poor diet, obesity, and associated chronic conditions in these vulnerable groups.

Because adolescents face increased exposure to targeted ads on social media, two US Senators introduced bipartisan legislation in 2019 to update the Children's Online Privacy and Protection Act, which regulates how companies collect and use online data from children (age <13 years) and stipulates the extent to which children can be targeted with online ads.^28^ The Senators' proposal aimed to expand protections (e.g., further limit data collected from children online), apply the protections to adolescents up to age 15 years, and eliminate racially targeted marketing practices online (e.g., via geo-tagging features).^29^

But there is a critical gap in our understanding of the online landscape of racially targeted food advertising: no studies, to our knowledge, have examined whether there is an association between racially targeted marketing practices and the number of adolescents who engage with advertisements on social media. Objective data on Black and Hispanic followers of food and beverage companies are critical for determining the extent to which policies should regulate online advertising that targets adolescents of color.

To address this gap in the literature, we analyzed social media data from Instagram and Twitter. This study aimed to: (1) quantify the percentage of Black, Hispanic, and White individuals who follow food and beverage brands; (2) compare whether unhealthy food brands and sugary drink brands have higher percentages of Black and Hispanic followers relative to all social media accounts; (3) determine whether sugary drink brands have more Black and Hispanic followers compared with low-calorie drink brands; and (4) examine the association between targeted marketing practices to youth of color and the percentage of Black, Hispanic, and White users who follow food and beverage brands.

## Methods

### Population

Our analysis draws from the 30 fast food, snack, and beverage brands with the highest ad expenditures in 2016.^30–32^ We chose to analyze only fast food, snack, and beverage brands because marketers disproportionately promote those types of products to adolescents and communities of color.^6^ In 2019, we purchased the data Demographics Pro on the demographic characteristics of social media users who followed the selected brands on Twitter and Instagram (data are not available on other social media platforms).

Including these two platforms is valuable for several reasons. Although YouTube is the most popular platform among adolescents, it is primarily used to view videos rather than post, share, and follow others users, making it less relevant to this study. And SnapChat, the second most popular social media site among adolescents, generates just $1.7 billion per year in advertising revenue.^33,34^ In comparison, Instagram generates $20 billion annually, and Twitter generates $3 billion annually—suggesting that ad exposure on these sites are high. Finally, although Twitter is only used by 32% of adolescents in the United States, that figure translates to roughly 8 million adolescents.^35,36^

Demographics Pro is a data analytics firm that uses proprietary algorithms to infer likely demographic characteristics of social media users based on their behavior on social media.^37–39^ The predictions rely on the low covariance of multiply amplified data signals. These data signals include the nature and strength of ties between users on Twitter and Instagram; the Twitter and Instagram accounts users follow; overall Twitter and Instagram usage; and the words and phrases used in posts. Big data methods, natural language processing, image analyses, and network theory are also used in the prediction of users' demographic characteristics.

To predict each demographic characteristic, Demographics Pro requires confidence of 95% or above. The firm evaluates their methods by iteratively testing their approach among established samples of between 10,000 and 200,000 social media users with verified demographic information, enabling more precise calibration of their methods. Using this methodology, Demographics Pro has profiled >300 million social media users. At the time of purchase, Demographics Pro had data on Instagram and Twitter users for 27 of the 30 brands, including 24 brands on Instagram and 19 brands on Twitter (Table 1).

**Table 1.** Total Numbers of Followers of Popular Food/Beverage Brands (*n*=27), by Platform and User Characteristics, 2019

Brand	Instagram				Twitter			
	Total followers (*n*)	Followers who have used platform >2 years [*n* (%)]	Followers who post 1–7 posts per week [*n *(%)]	Followers with geo-enabled network settings [*n* (%)]	Total followers (*n*)	Followers who have used platform >2 years [*n* (%)]	Followers who post 1–7 posts per week [*n* (%)]	Followers with geo-enabled network settings [*n* (%)]
Burger King	1,623,786	596,979 (36.8)	667,387 (41.1)	459,521 (28.3)	1,713,262	984,029 (57.5)	569,187 (33.2)	533,582 (31.1)
Chick-fil-A	1,256,639	812,392 (64.6)	570,680 (45.4)	373,141 (29.7)	958,494	677,337 (70.7)	296,902 (31)	370,218 (38.6)
Coca-Cola^a^	2,592,532	1,119,247 (43.2)	1,078,960 (41.6)	745,042 (28.7)	—	—	—	—
Coca-Cola Life	6,049	2,818 (46.6)	2,675 (44.2)	1,539 (25.4)	37,968	28,474 (75)	14,182 (37.4)	15,031 (39.6)
Coke Zero	98,742	29,992 (30.3)	38,760 (39.3)	13,886 (14.1)	253,914	238,469 (93.9)	86,676 (34.1)	967,52 (38.1)
Dairy Queen	473,337	241,847 (51.1)	206,677 (43.7)	87,300 (18.4)	477,430	426,219 (89.3)	152,802 (32)	179,847 (37.7)
Dasani Water^b^	—	—	—	—	14,327	12,588 (87.9)	4,397 (30.7)	5,260 (36.7)
Denny's Diner^b^	—	—	—	—	520,034	396,021 (76.2)	158,581 (30.5)	205,970 (39.6)
Diet Coke	79,181	50,715 (64.1)	39,866 (50.3)	21,025 (26.6)	305,944	295,673 (96.7)	101,990 (33.3)	129,283 (42.3)
Dr. Pepper^a^	536,521	234,595 (43.7)	221,669 (41.3)	104,732 (19.5)	—	—	—	—
Fanta	517,501	168,984 (32.6)	202,499 (39.1)	64,924 (12.5)	157,722	146,459 (92.9)	54,513 (34.6)	51,222 (32.5)
Gatorade	1,357,038	512,855 (43.9)	382,004 (32.7)	310,416 (26.6)	331,396	314,292 (94.9)	106,706 (32.2)	133,220 (40.2)
KFC^a^	1,357,038	481,070 (35.4)	514,942 (37.9)	312,852 (23.1)	—	—	—	—
McDonald's^a^	3,342,259	1,366,449 (40.9)	1,324,871 (39.6)	846,327 (25.3)	—	—	—	—
Monster Energy	5,027,096	2,520,385 (50.1)	1,813,475 (36.1)	1,650,094 (32.8)	3,198,430	1,819,119 (56.9)	1,154,135 (36.1)	914,863 (28.6)
Mountain Dew	425,378	214,414 (50.5)	157,461 (37)	110,123 (25.9)	564,512	470,156 (83.2)	991,810 (33.1)	1,037,268 (34.6)
Oreo	2,506,211	1,070,202 (42.7)	1,080,879 (43.1)	685,348 (27.3)	834,516	567,998 (68.1)	268,371 (32.2)	289,157 (34.6)
Pepsi^a^	1,438,122	534,061 (37.2)	571,168 (39.7)	276,739 (19.2)	—	—	—	—
Pizza Hut^a^	1,527,842	597,607 (39.1)	620,068 (40.6)	375,862 (24.6)	—	—	—	—
Red Bull	10,293,957	5,952,480 (57.8)	3,905,733 (37.9)	4,297,830 (41.8)	2,101,969	1,933,109 (92)	743,263 (35.4)	865,793 (41.2)
Smart Water	48,841	34,260 (70.1)	24,084 (49.3)	13,371 (27.4)	5,493	4,992 (90.8)	1,823 (33.2)	2,453 (44.7)
Sprite	869,636	343,005 (39.5)	335,278 (38.6)	132,499 (15.2)	284,233	260,738 (91.8)	92,532 (32.6)	104,338 (36.7)
Starbucks^a^	17,425,064	9,655,053 (55.4)	7,782,208 (44.7)	6,081,522 (34.9)	—	—	—	—
Subway	1,030,818	440,138 (42.7)	419,724 (40.7)	165,710 (16.1)	2,361,855	1,518,739 (64.3)	782,893 (33.1)	740,445 (31.4)
Taco Bell^a^	1,274,017	758,805 (59.5)	537457 (42.2)	409673 (32.2)	—	—	—	—
Vitamin Water^b^	—	—	—	—	161,000	159,723 (99.2)	53,840 (33.4)	70,916 (44)
Wendy's	834,654	400,339 (47.9)	357,171 (42.8)	147,687 (17.7)	3,000,678	2,088,191 (69.6)	991,810 (33.1)	1,037,268 (34.6)
Total	55,942,259	28,138,692	22,855,696	17,687,163	17,283,177	12,342,326	6,626,413	6,782,886

^a^ Instagram only.

^b^ Twitter only.

### Measures

We used the University of Connecticut Rudd Center's 2019 report on targeted food advertising to characterize the degree to which the 27 selected brands targeted Black consumers.^8^ The report provides total TV spending as well as a Black:White targeted ratio for children and adolescents, including separate measures for 2–5 years, 6–11 years, and 12–17 years. Data were available for 9 of the 27 brands we selected (Table 2). The targeted ratios are calculated by dividing the ratio of TV ad exposure for Black children/adolescents by the ratio of TV ad exposure to White children/adolescents. To account for racial differences in time spent viewing ads, this value is then divided by the ratio of TV viewing times. For example, a Black:White targeted ratio of 2.00 indicates that Black children/adolescents viewed 100% more TV ads for a specific brand compared with White children/adolescents, TV viewing being equal.

**Table 2.** Racial Demographics of Followers and Targeted Marketing Characteristics of Popular Food/Beverage Brands (*n*=27), 2019

Brand	Instagram			Twitter			Black:White targeted ratio (2–5 years)^c^	Black:White targeted ratio (6–11 years)^c^	Black:White targeted ratio (12–17 years)^c^
	Black followers [*n* (%)]	White followers [*n* (%)]	Hispanic followers [*n* (%)]	Black followers [*n* (%)]	White followers [*n* (%)]	Hispanic followers [*n* (%)]			
Burger King	116,510 (16.2)	503,872 (70)	94,748 (13.2)	154,785 (12.2)	977,618 (77.1)	128,258 (10.1)	2.04	2.08	2.08
Chick-fil-A	137,234 (11.6)	957,270 (80.9)	83,161 (7)	104,430 (11.5)	727,644 (79.9)	73,865 (8.1)	—	—	—
Coca-Cola^a^	133,742 (12.7)	806,873 (76.5)	107,637 (10.2)	—	—	—	1.94	1.94	1.94
Coca-Cola Life	116 (4.7)	2,239 (90.1)	116 (4.7)	4,159 (11.6)	28,937 (80.4)	2,680 (7.4)	1.81	1.70	1.70
Coke Zero	3,892 (11.1)	27,486 (78.6)	3,412 (9.8)	17,381 (9.7)	146,411 (81.9)	14,016 (7.8)	2.25	2.09	2.09
Dairy Queen	37,494 (10.1)	301,193 (81.1)	31,452 (8.5)	39,901 (9.3)	350,886 (82.2)	34,514 (8.1)	1.48	1.40	1.40
Dasani Water^b^	—	—	—	1,090 (8.1)	11,648 (86.4)	679 (5)	—	—	—
Denny's Diner^b^	—	—	—	24,242 (5.1)	409,546 (86.9)	34,024 (7.2)	—	—	—
Diet Coke	2,653 (4.9)	48,381 (89.8)	2,582 (4.8)	21,028 (10)	174,613 (82.7)	14,226 (6.7)	1.64	1.71	1.71
Dr. Pepper^a^	44,390 (12)	289,282 (78.4)	32,996 (8.9)	—	—	—	—	—	—
Fanta	30,350 (19.3)	103,329 (65.7)	21,578 (13.7)	16,513 (14.2)	86,729 (74.6)	12,266 (10.6)	—	—	—
Gatorade	114,690 (13.2)	654,796 (75.5)	93,600 (10.8)	38,731 (13.5)	219,659 (76.5)	27,216 (9.5)	2.37	2.29	2.29
KFC^a^	68,369 (13.9)	357,761 (72.7)	60,772 (12.3)	—	—	—	1.90	1.97	1.97
McDonald's^a^	224,200 (15.7)	1,051,573 (73.4)	145,857 (10.2)	—	—	—	1.81	1.87	1.87
Monster Energy	217,044 (10.4)	1,734,072 (83)	129,465 (6.2)	76,278 (7.8)	776,914 (79.9)	114,173 (11.7)	—	—	—
Mountain Dew	29,746 (9.8)	241,449 (79.8)	29,567 (9.8)	43,099 (8.8)	403,835 (82.9)	38,375 (7.9)	2.31	2.30	2.30
Oreo	98,959 (11)	718,434 (79.8)	75,023 (8.3)	47,340 (8.2)	476,278 (82)	53,056 (9.1)	2.00	2.14	2.14
Pepsi^a^	91,620 (12.7)	548,121 (76)	75,977 (10.5)	—	—	—	2.03	1.97	1.97
Pizza Hut^a^	114,106 (14.9)	547,312 (71.5)	98,526 (12.9)	—	—	—	1.93	1.94	1.94
Red Bull	369,343 (9)	3,503,515 (85)	225,593 (5.5)	73,563 (8.1)	742,387 (82.1)	83,525 (9.2)	—	—	—
Smart Water	4,892 (12.5)	30,523 (78.2)	3,310 (8.5)	36 (7.9)	391 (86.8)	21 (4.6)	—	—	—
Sprite	152,969 (30.7)	279,637 (56.1)	60,542 (12.2)	49,848 (22.7)	145,075 (66)	23,466 (10.7)	2.98	3.11	3.11
Starbucks^a^	864,715 (10.1)	7,103,593 (83.1)	505,539 (5.9)	—	—	—	—	—	—
Subway	68,443 (14.3)	350,306 (73)	57,372 (12)	243,740 (13.1)	1,434,571 (77.1)	172,432 (9.3)	1.93	1.94	1.94
Taco Bell^a^	—	—	—	30,319 (22.9)	882,60 (66.7)	12,867 (9.7)	2.11	2.20	2.20
Vitamin Water^b^	99,924 (9.2)	883,183 (81.2)	91,930 (8.5)	—	—	—	2.82	2.65	2.65
Wendy's	95,035 (15.8)	443,737 (73.6)	60,637 (10.1)	294,955 (11.5)	2,013,950 (78.7)	234,685 (9.2)	2.10	2.13	2.13
Total	3,120,436	21,487,937	2,091,392	1,281,438	9,215,352	1,074,344			

^a^ Instagram only.

^b^ Twitter only.

^c^ Data Source: UConn Rudd Center.^19^ They calculated the ratios by dividing the ratio of TV ad exposure for Black youth versus White youth by the ratio of TV viewing times.

Data on spending and disproportionate exposure to social media advertising were not available for social media, so we used spending on TV ads and disproportionate exposure to TV advertising as proxies. Without such measures for adult social media users, we also used targeted ratios for Black children/adolescents as proxies for targeted advertising to Black adults. The report, however, does not include a Hispanic:White ratio for children and adolescents. They include data on food and beverage company expenditures on Spanish-language television, which we included in analyses to examine the relationship between targeted marketing expenditures and number of Hispanic followers in our sample.

Our primary outcome was the percentage of social media users who followed the selected food/beverage brands on each platform, by racial demographic subgroup. We also calculated the percentage of followers by race/ethnicity for (1) fast food brands, (2) all beverage brands, (3) sugary drink brands, and (4) low-calorie drink brands (Table 2). We classified both zero-calorie beverages and beverages with artificial sweeteners as low-calorie drink brands. We reported the mean (standard deviation) of the percentage of followers of the selected brands, as well as user posting frequency, length of time since account creation, and user's geo-enabled settings.

### Statistical analysis

Using a one-sample *t*-test, we examined whether the mean percentage of followers of the selected food and beverage brands was statistically significantly different from the mean percentage of followers of any account on Instagram or Twitter, by racial demographic subgroup. Using an independent samples *t*-test, we also tested whether the mean percentage of followers of the sugary drink brands was statistically significantly different compared with the mean percentage of followers of the low-calorie drink brands. We used a linear regression model to examine the association between spending on TV advertising and the Black:White-targeted ratios of the selected food/beverage brands with the racial demographic characteristics of followers, by social media platform.

Finally, for Hispanic targeting, we used a linear regression model to examine the association between ad expenditures on Spanish-language television and ethnic demographic characteristics of followers, by social media platform. Statistical significance was defined at the *α*=0.05 level. We used Stata version 15.1 (StataCorp LLC, College Station, TX) for all analyses.^40^ We downloaded and analyzed the data in January 2019.

## Results

There were an estimated total of 55.9 million users who followed the 27 food/beverage brands on Instagram, and 17.3 million users who followed the brands on Twitter in January 2019, including users who followed multiple accounts on both Instagram and Twitter (Table 1). An estimated total of 17.7 million and 6.8 million followers of the selected brands had geo-enabled network settings on Instagram and Twitter, respectively.

### Followers of food/beverage brands versus any social media account by race/ethnicity

Among all Instagram users, an estimated total of 3.1 million Black, 2.1 million Hispanic, and 21.5 million White individuals followed the selected food/beverage brands; and among all Twitter users, an estimated total of 1.3 million Black, 1.1 million Hispanic, and 9.2 million White individuals followed the selected brands (Table 2). Sprite (30.7%) and Fanta (19.3%) had the highest percentage of Black followers on Instagram relative to other beverage brands (<14%). Fanta (13.7%) also had the highest percentage of Hispanic followers on Instagram, along with Burger King (13.2%), Pizza Hut (12.9%), KFC (12.3%), and Sprite (12.2%).

In contrast, Coca-Cola Life (90.1%) and Diet Coke (89.8%) had the highest percentage of White followers relative to other beverage brands (<85%). On Twitter, Taco Bell (22.9%) and Sprite (22.7%) had the highest percentages of Black followers relative to other brands, whereas Monster Energy Drink (11.7%) and Fanta (10.6%) had the highest percentage of Hispanic followers (Table 2).

We found that the percentage of White followers of the selected food/beverage brands (77.2%) was lower than the percentage of White individuals who followed any account on Instagram (81.1%) (*p*=0.02) (Table 3). We observed a similar finding for White followers of fast food brands relative to all social media accounts on Instagram (75.3% vs. 81.1%; *p*=0.01). In contrast, the percentage of Black followers of the selected brands (12.7%) was higher than the percentage of Black individuals who followed any account on Instagram (7.8%) (*p*<0.001). We observed a similar pattern for fast food brands (13.5% vs. 7.8%; *p*<0.001). The percentage of Hispanic followers of the selected brands (8.5%) was also higher than the percentage of Hispanic individuals who followed any account on Twitter (7.6%) (*p*=0.04), and even higher for sugary drink brands (9.9%).

**Table 3.** Comparison of Demographics of Followers of Popular Food/Beverage Brands (*n*=27) Versus All Users, 2019

	All brands	*p*^a^	Fast food brands	*p*^a^	Drink brands	*p*^a^	Sugary drink brands	*p*^a^	Low-calorie drink brands	*p*^a^	Average on platform
Twitter (% followers)											
Race (United States only)											
White	79.5	0.54	80.3	0.34	78.9	0.92	77^b^	0.54	80.8^b^	0.51	78.7
Black	11.4	0.12	10.5	0.08	12.1	0.54	12.5	0.81	11.7	0.57	13.1
Hispanic	8.5	0.04	8.7	0.06	8.4	0.24	9.9^b^	0.01	6.9^b^	0.39	7.6
Instagram (% followers)											
Race (United States only)											
White	77.2	0.02	75.3	0.01	77.9	0.23	75.1	0.08	84.2	0.42	81.1
Black	12.7	<0.001	13.5	<0.001	12.5	0.02	14.4	0.02	8.3	0.82	7.8
Hispanic	9.4	0.07	10.5	0.87	8.9	0.08	9.8	0.48	7.0	0.08	10.4

^a^ The mean percentage of followers of the group of brands was statistically significantly different (*p*<0.05) from the mean percentage of all Twitter/Instagram users using a one-sample *t*-test.

^b^ The mean percentage of followers of sugary drink brands was statistically significantly different (*p*<0.05) than the mean percentage of followers of low-calorie drink brands using an independent samples *t*-test.

We did not, however, find statistically significant differences in the percentages of White and Black followers of the selected brands relative to all social media accounts on Twitter.

### Followers of sugary drink brands versus low-calorie drink brands by race/ethnicity

On Twitter, the percentage of Hispanic followers of sugary drink brands (9.9%) was higher than the percentage Hispanic followers of low-calorie drink brands (6.9%) (*p*<0.001) (Table 3), whereas the percentage of White followers of sugary drink brands (77.0%) was lower than the percentage of White followers of low-calorie drink brands (80.8%) (*p*=0.03). The direction of differences was similar using data from Instagram, but not statistically significant. We did not observe statistically significant differences in the percentage of Black followers of sugary drink brands compared with low-calorie drink brands on either platform.

### Associations between racially targeted marketing practices and the percentage of followers by race/ethnicity

When examining the relationship between racially targeted advertising and follower demographics, we found that a higher Black:White child-targeted ratio (2–5 years) was positively associated with the percentage of Black followers of the selected brands on Twitter (β=9.0; *p*=0.001); and a higher Black:White child-targeted ratio (2–5 years) was negatively associated with the percentage of White followers of the selected brands on Twitter (β=−10.8; *p*=0.002) (Table 4). Findings for targeted ratios for older children (6–11 years) and adolescents (12–17 years) were similar in direction and magnitude for Black and White followers. The direction and magnitude of the results using Instagram data were also similar for all racially targeted ratio measures.

**Table 4.** Associations Between Targeted Marketing Ratios and Demographics of Followers of Popular Food/Beverage Brands (*n*=27), 2019

	Total TV spending (in $10 million)	*p*	Spanish-language TV spending (in $10 million)	*p*	Black:White targeted ratio (2–5 years)^a^	*p*	Black:White targeted ratio (6–11 years)^a^	*p*	Black:White targeted ratio (12–17 years)^a^	*p*
Twitter (% followers)										
White	0.3	0.85	—	—	−10.8	0.001^b^	−10.4	0.002^b^	−9.9	<0.001^b^
Black	0.3	0.65	—	—	9.0	0.002^c^	8.4	0.004^c^	8.0	0.001^c^
Hispanic	0.1	0.29	0.002	0.45	—	—	—	—	—	—
Instagram (% followers)										
White	−1.1	0.38	—	—	−15.4	0.004^b^	−15.2	0.002^b^	−14.2	0.001^b^
Black	0.5	0.56	—	—	12.1	0.002^c^	11.4	0.00^c^	0.5	<0.001^c^
Hispanic	0.5	0.17	0.0005	0.88	—	—	—	—	—	—

^a^ The targeted ratios are calculated by dividing the ratio of TV ad exposure for Black youth versus White youth by the ratio of TV viewing times.

^b^ These findings show that a higher degree of racially targeted marketing (i.e., more TV ad exposure per TV viewing time) per brand was associated with disproportionately lower percentage of White followers per brand.

^c^ These findings show that a higher degree of racially targeted marketing (i.e., more TV ad exposure per TV viewing time) per brand was associated with a disproportionately higher percentage of Black followers per brand.

We did not, however, observe a relationship between TV spending and the percent of Black, Hispanic, or White followers of the selected brands using data from either platform; and we did not observe a relationship between Spanish-language TV spending and the percent of Hispanic followers on either platform.

## Discussion

These data show that the 27 food and beverage brands in our sample maintained a total of 73.2 million followers on Twitter and Instagram as of 2019, including some users who may follow multiple accounts on both platforms. This study provides the first evidence, to our knowledge, demonstrating that popular food and beverage brands have a disproportionately higher percentage of Black and Hispanic followers relative to users who follow other social media accounts.

Our study also provides new evidence to show that racially targeted advertising ratios are associated with a higher percentage of Black followers—and a lower percentage of White followers—of popular food and beverage brands (Table 4). Sprite (140 calories per serving) and Fanta (160 calories per serving), for example, are sugary beverage brands that target Black youth more than zero-calorie beverage brands like Diet Coke and Dasani Water. All three brands are owned by the same parent company (Coca-Cola) and yet 22.7% of Sprite's Twitter followers are Black, whereas 10.0% of Diet Coke's and 8.1% of Dasani's Twitter followers are Black. Companies like Coca-Cola should promote a balance of healthy beverages to all consumers, rather than disproportionately targeting sugary drinks to communities that experience high rates of diabetes and obesity.^41^

Nearly half (40.0%) of social media followers in our sample had geo-enabled network settings, which facilitates companies' use of their location for targeted marketing. Although geo-targeting may be convenient for individuals who receive coupons to nearby stores, such targeting holds the potential to exacerbate health disparities if companies promote coupons for unhealthy products to communities that experience high rates of obesity and diabetes.^42^ In response to growing concerns about the unique features of social media marketing, two U.S. Senators introduced bipartisan legislation in March 2019 to update the Children's Online Privacy and Protection Act, including restricting companies' use of geo-targeting and racially targeted advertising.^29^ Our data reinforce the need for such an expansion.

These findings build on previous research that examined website traffic of food and beverage brands that target Black and Hispanic youth.^43^ The authors used ComScore data to report that Hispanic youth were more likely to visit websites for food and beverages that target Hispanic consumers than non-Hispanic youth.^43^ Our findings also corroborate previous research showing that non-Hispanic Black and less-acculturated Hispanic adolescents were more likely than non-Hispanic White adolescents to engage with food and beverage brands on social media.^44^ Although we did not assess why some Black and Hispanic social media users choose to follow food and beverage brands, other studies found that Black and Hispanic consumers report feeling valued by food and beverage companies that target members of their racial/ethnic group.^45^

We also found that sugary drink brands have higher percentages of Black and Hispanic followers than low-calorie drink brands on Twitter, which may be driven by racially targeted marketing practices of sugary drink brands. The opposite pattern was true for low-calorie drink brands, which had higher percentages of White followers than sugary drink brands. Overall in the United States, National Health and Nutrition Examination Survey (NHANES) data indicate that, among a sample of 16,492 youth, ∼19% drank low-calorie sweetened beverages.^46^ Given that Black and Hispanic youth and adults experience high rates of obesity and diabetes relative to other racial/ethnic groups, it is critical to develop policy interventions that address unhealthy food ads that target adolescents of color.

Our study had several limitations. We were not able to identify any sources of data that report social media ad expenditures made by food and beverage brands, but we posit that measures of targeted marketing on television are adequate proxies. This is because we expect relative differences in expenditures on racially targeted advertising on TV and social media to be consistent across brands, although absolute expenditures may differ within brands. For example, greater expenditures on racially targeted advertising for Sprite versus Diet Coke on TV likely translates to a similar difference in ad expenditures for Sprite versus Diet Coke on social media. In the future, academic institutions may consider working with commercial vendors who specialize in aggregating digital advertising data and optimizing the collection of such data for research opportunities (e.g., including more data on Hispanic consumers and more detailed data on advertising expenditures).

Another limitation is that Demographics Pro did not have data on all 27 brands across Instagram and Twitter, which limited our ability to compare Instagram and Twitter followers' demographics. We also lacked data to characterize the extent to which the brands in our sample targeted Hispanic consumers, although we used data on food and beverage company expenditures on Spanish-language television as a proxy, and lacked targeted ratios for adult social media users. Furthermore, the data from Demographics Pro are inferences based on consumers' social media presence and usage, which may not accurately reflect actual demographics of users. Despite these limitations, our study is the first to characterize the link between brands' targeted food marketing practices and the demographics of social media users who followed such brands.

## Conclusions

In our sample, food and beverage brands that heavily target youth of color have a disproportionately higher percentage of Black followers—and a disproportionately lower percentage of White followers—compared with brands that do not heavily target youth of color. The positive association between racially targeted marketing and percentage of Black social media followers is concerning for public health nutrition. Exposure to food advertising is a major driver of poor diet among youth, and Black and Hispanic youth are disproportionately targeted with the least healthy food products.^8,14^ Such targeting is concerning given our findings show that food and beverage brands have a disproportionately high percentage of Black and Hispanic followers relative to White followers.

In addition, sugary drink brands have a higher percentage of Hispanic followers relative to low-calorie drink brands. Given that many of these brands promote sugary drinks and unhealthy fast food products to Black and Hispanic children and adolescents, it is possible that food marketing on social media contributes to poor dietary habits and disparities in obesity risk among youth of color.

## Abbreviations Used

NHANES: National Health and Nutrition Examination Survey
NIH: National Institutes of Health
TV: television

## References

[1] Cairns G, Angus K, Hastings G, et al. Systematic reviews of the evidence on the nature, extent and effects of food marketing to children. A retrospective summary. Appetite. 2013;62:209–215 22561190 10.1016/j.appet.2012.04.017

[2] Halford JCG, Boyland EJ, Hughes G, et al. Beyond-brand effect of television (TV) food advertisements/commercials on caloric intake and food choice of 5–7-year-old children. Appetite. 2007;49:263–267 17258351 10.1016/j.appet.2006.12.003

[3] Halford JC, Boyland EJ, Hughes GM, et al. Beyond-brand effect of television food advertisements on food choice in children: the effects of weight status. Public Health Nutr. 2008;11:897–904 18005487 10.1017/S1368980007001231

[4] Halford JCG, Gillespie J, Brown V, et al. Effect of television advertisements for foods on food consumption in children. Appetite. 2004;42:221–225 15010186 10.1016/j.appet.2003.11.006

[5] Harris JL, Bargh JA, Brownell KD. Priming effects of television food advertising on eating behavior. Health Psychol. 2009;28:404–413 19594263 10.1037/a0014399 PMC2743554

[6] Institute of Medicine, Board on Children, Youth, and Families, Food and Nutrition Board, Committee on Food Marketing and the Diets of Children and Youth. Food Marketing to Children and Youth: Threat or Opportunity? Washington, DC: National Academies Press, 2006, 536 p

[7] Powell LM, Wada R, Kumanyika SK. Racial/ethnic and income disparities in child and adolescent exposure to food and beverage television ads across the U.S. media markets. Health Place. 2014;29:124–131 25086271 10.1016/j.healthplace.2014.06.006 PMC4501500

[8] Harris JL, Frazier W III, Kumanyika S, et al. Increasing disparities in unhealthy food advertising targeted to Hispanic and Black youth [Internet]. Rudd Center for Food Policy and Obesity. 2019. Available at http://uconnruddcenter.org/files/Pdfs/TargetedMarketingReport2019.pdf Accessed 84, 2020

[9] Grier SA, Kumanyika S. Targeted marketing and public health. Annu Rev Public Health. 2010;31:349–369 20070196 10.1146/annurev.publhealth.012809.103607

[10] Harris JL, Shehan C, Gross R, et al. Food advertising targeted to Hispanic and Black youth: contributing to health disparities. Rudd Center Food Policy Obes 2015. https://uconnruddcenter.org/wp-content/uploads/sites/2909/2020/09/272-7-Rudd_Targeted-Marketing-Report_Release_0811151.pdf Accessed 84, 2020.

[11] Use of internet, social media, digital devices plateau in US [Internet]. Available at https://www.pewresearch.org/fact-tank/2018/09/28/internet-social-media-use-and-device-ownership-in-u-s-have-plateaued-after-years-of-growth/ Accessed 84, 2020

[12] Commission FT, Others. A Review of Food Marketing to Children and Adolescents: Follow-Up Report. Washington, DC: Offıce US Government Printing Office, 2012

[13] McClure AC, Tanski SE, Gilbert-Diamond D, et al. Receptivity to television fast-food restaurant marketing and obesity among U.S. youth. Am J Prev Med. 2013;45:560–568 24139768 10.1016/j.amepre.2013.06.011 PMC3934414

[14] Scully M, Wakefield M, Niven P, et al. Association between food marketing exposure and adolescents' food choices and eating behaviors. Appetite. 2012;58:1–5 22001023 10.1016/j.appet.2011.09.020

[15] Story M, Neumark-Sztainer D, French S. Individual and environmental influences on adolescent eating behaviors. J Am Diet Assoc. 2002;102(3 Suppl.):S40–S51 11902388 10.1016/s0002-8223(02)90421-9

[16] Anderson M, Jiang J. Teens, Social Media & Technology. Washington, DC: Pew Internet & American Life Project, 2018

[17] Ignatius A. Shaking Things up at Coca-Cola [Internet]. Harvard Business Review. 2011. Available at https://hbr.org/2011/10/shaking-things-up-at-coca-cola Accessed 84, 2020

[18] Bragg MA, Pageot YK, Amico A, et al. Fast food, beverage, and snack brands on social media in the United States: an examination of marketing techniques utilized in 2000 brand posts. Pediatr Obes. 2020;15:e12606 31875654 10.1111/ijpo.12606 PMC9743983

[19] Montgomery K, Chester J, Nixon L, et al. Big Data and the transformation of food and beverage marketing: undermining efforts to reduce obesity? Crit Public Health. 2019;29:110–117

[20] Vandevijvere S, Sagar K, Kelly B, et al. Unhealthy food marketing to New Zealand children and adolescents through the internet. N Z Med J. 2017;130:32–43 28207723

[21] Freeman B, Kelly B, Baur L, et al. Digital junk: food and beverage marketing on Facebook. Am J Public Health. 2014s;104:e56–e64 10.2105/AJPH.2014.302167 PMC4232106 25322294

[22] Montgomery KC, Chester J, Grier SA, et al. The new threat of digital marketing. Pediatr Clin North Am. 2012;59:659–675, viii. 22643172 10.1016/j.pcl.2012.03.022

[23] Why Do People Follow Brands On Social Media? [Internet]. 2020. Available at https://www.marketingcharts.com/digital/social-media-113405 Accessed 84, 2020

[24] Lin H-C, Swarna H, Bruning PF. Taking a global view on brand post popularity: six social media brand post practices for global markets. Bus Horiz. 2017;60:621–633

[25] Beukeboom CJ, Kerkhof P, de Vries M. Does a virtual like cause actual liking? How following a brand's Facebook updates enhances brand evaluations and purchase intention. J Interact Mark. 2015;32:26–36

[26] de Vries L, Gensler S, Leeflang PSH. Popularity of Brand Posts on Brand Fan Pages: an Investigation of the Effects of Social Media Marketing. J Interact Mark. 2012;26:83–91

[27] Saboo AR, Kumar V, Ramani G. Evaluating the impact of social media activities on human brand sales. Int J Res Mark. 2016;33:524–541

[28] Commission FT, Others. Children's online privacy protection act of 1998, 1998. Available at https://www.ftc.gov/enforcement/rules/rulemaking-regulatory-reform-proceedings/childrens-online-privacy-protection-rule Accessed 84, 2020

[29] Senator Markey Statement on FTC Effort to Update Children's Privacy Rules [Internet]. 2019. Available at https://www.markey.senate.gov/news/press-releases/senator-markey-statement-on-ftc-effort-to-update-childrens-privacy-rules Accessed 84, 2020

[30] Harris JL, Schwartz MB, Shehan C, et al. Snack FACTS 2015: evaluating Snack Food Nutrition and Marketing to Youth [Internet]. Connecticut: UConn Rudd Center for Food Policy & Obesity. 2015. Available at www.uconnruddcenter.org/files/Pdfs/SnackFACTS_2015_Fulldraft03.pdf Accessed 84, 2020

[31] Harris JL, Schwartz MB, LoDolce M, et al. Sugary drink FACTS 2014: some progress but much room for improvement in marketing to youth. Rudd Center for Food Policy and Obesity: New Haven, CT [Internet]. 2014. Available at www.sugarydrinkfacts.org/resources/sugarydrinkfacts_report.pdf Accessed 84, 2020

[32] Harris JL, Schwartz MB, Munsell CR, et al. Fast food FACTS 2013: measuring progress in nutrition and marketing to children and teens. New Haven, CT: Yale Rudd Center for Food Policy and Obesity [Internet]. 2013. Available at www.fastfoodmarketing.org/media/fastfoodfacts_report.pdf Accessed 84, 2020

[33] Pires F, Masanet M-J, Scolari CA. What are teens doing with YouTube? Practices, uses and metaphors of the most popular audio-visual platform. Null. 2019;1–17

[34] sec-show.pdf. Available at https://s25.q4cdn.com/442043304/files/annual/sec-show.pdf Accessed 84, 2020

[35] Simon E. How Instagram Makes Money [Internet]. 2020. Available at https://www.investopedia.com/articles/personal-finance/030915/how-instagram-makes-money.asp Accessed 84, 2020

[36] POP1 Child population: Number of children (in millions) ages 0–17 in the United States by age, 1950–2019 and projected 2020–2050 [Internet]. Available at https://www.childstats.gov/americaschildren/tables/pop1.asp Accessed 84, 2020

[37] Cavazos-Rehg PA, Krauss MJ, Sowles S, et al. A content analysis of depression-related Tweets. Comput Human Behav. 2016;54:351–357 26392678 10.1016/j.chb.2015.08.023 PMC4574287

[38] Cavazos-Rehg PA, Sowles SJ, Krauss MJ, et al. A content analysis of tweets about high-potency marijuana. Drug Alcohol Depend. 2016;166:100–108 27402550 10.1016/j.drugalcdep.2016.06.034 PMC4983477

[39] Metwally O, Blumberg S, Ladabaum U, et al. Using social media to characterize public sentiment toward medical interventions commonly used for cancer screening: an observational study. J Med Internet Res. 2017;19:e200 28592395 10.2196/jmir.7485 PMC5480009

[40] StataCorp. Stata Statistical Software: Release 15. College Station, TX: StataCorp LLC, 2017

[41] Hales CM, Carroll MD, Fryar CD, et al. Prevalence of Obesity Among Adults and Youth: United States, 2015–2016. National Center for Health Statistics; 2017 Oct. Report No.: 288 29155689

[42] Chen Y, Li X, Sun M. Competitive mobile geo targeting. Mark Sci. 2017;36:666–682

[43] Hyary M, Harris JL. Hispanic youth visits to food and beverage company websites. Health Equity. 2017;1:134–138 30283841 10.1089/heq.2016.0026 PMC6071883

[44] Fleming-Milici F, Harris JL. Adolescents' engagement with unhealthy food and beverage brands on social media. Appetite. 2020;146:104501 31669579 10.1016/j.appet.2019.104501

[45] DiSantis KI, Kumanyika S, Carter-Edwards L, et al. Sensitizing black adult and youth consumers to targeted food marketing tactics in their environments [Internet]. Int J Environ Res Public Health. 2017;14:1316 10.3390/ijerph14111316 PMC5707955 29109377

[46] Sylvetsky AC, Jin Y, Clark EJ, et al. Consumption of Low-Calorie Sweeteners among Children and Adults in the United States. J Acad Nutr Diet. 2017;117:441.e2–448.e2. 28087414 10.1016/j.jand.2016.11.004 PMC6176710

